# Towards hydrophobic carminic acid derivatives and their incorporation in polyacrylates

**DOI:** 10.1098/rsos.172399

**Published:** 2018-07-04

**Authors:** Luca Gabrielli, Davide Origgi, Giuseppe Zampella, Luca Bertini, Simone Bonetti, Gianfranco Vaccaro, Francesco Meinardi, Roberto Simonutti, Laura Cipolla

**Affiliations:** 1 Department of Biotechnology and Biosciences, University of Milan-Bicocca, P.zza della Scienza 2, 20126 Milan, Italy; 2 Department of Material Sciences, University of Milan-Bicocca, Via Cozzi 55, 20125 Milan, Italy

**Keywords:** biocolourants, carminic acid, polyacrylates, photoluminescence, density functional theory

## Abstract

Carminic acid, a natural hydrophilic dye extensively used in the food and cosmetic industries, is converted in hydrophobic dyes by acetylation or pivaloylation. These derivatives are successfully used as biocolourants for rubber objects. In this paper, spectroscopic properties of the carminic acid derivatives in dimethyl sulfoxide and in polybutylacrylate are studied by means of photoluminescence and time-resolved photoluminescence decays, revealing a hypsochromic effect due to the presence of bulky substituents as the acetyl or pivaloyl groups. Molecular mechanics and density functional theory calculations confirm the disruption of planarity between the sugar ring and the anthraquinoid system determined by the esterification.

## 1. Introduction

**Figure 1.**
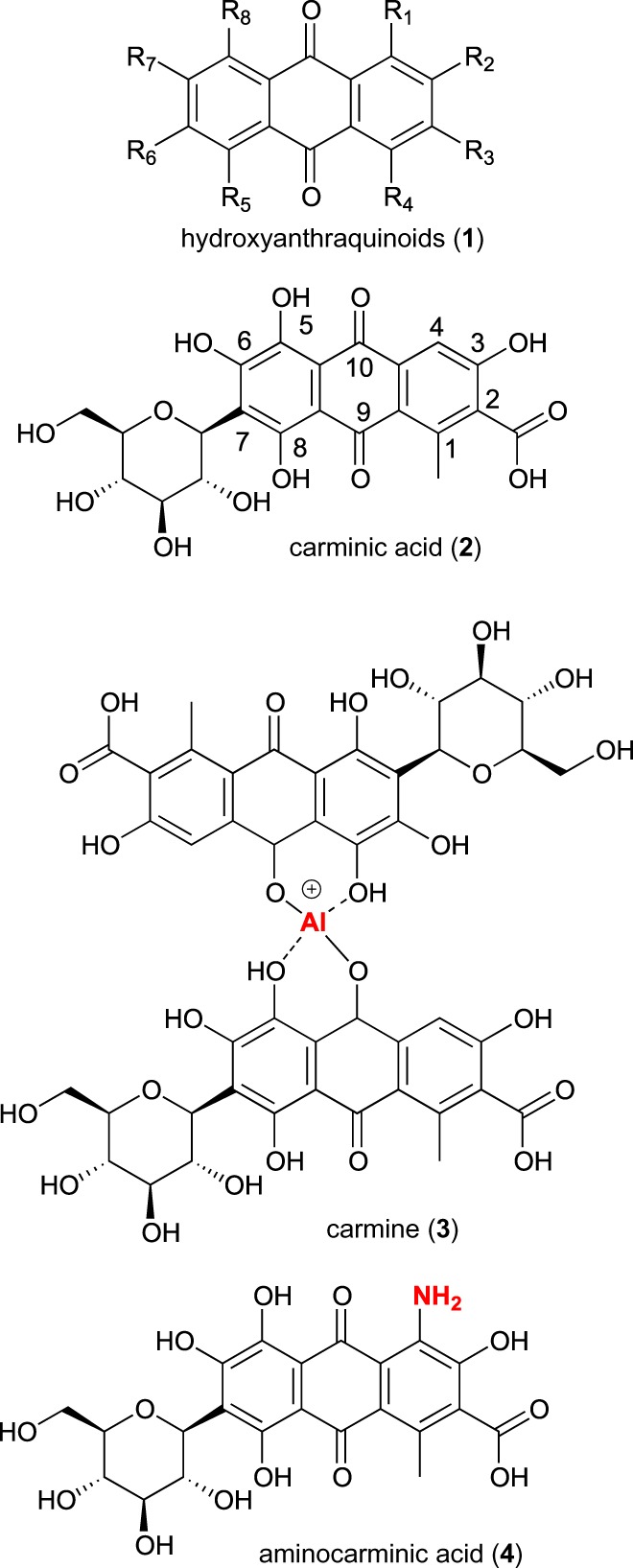
General structure of HAQN (**1**) and structures of carminic acid (**2**), carmine (**3**) and 4-aminocarminic acid (**4**).

Natural pigments comprise a wide range of natural products belonging to different classes [1] such as carotenoids, anthocyanins, chlorophylls, melanins, betalains and hydroxyanthraquinoids. Hydroxyanthraquinoids **1** (HAQN, figure 1) have found wide application [2] in cosmetic manufacturing, textile dyeing [3], as food biocolourants [4,5], or more recently, in coordination chemistry applications such as self-assembled systems, coordination polymers, metal-organic compounds, chemosensors [4] and dye-sensitized solar cells [6]. All HAQN dyes are highly hydroxylated molecules, featured with extended intra- and intermolecular hydrogen bonding responsible for their sparingly pH-dependent solubility in water. In addition, they are usually insoluble in apolar systems; as a consequence of solubility issues, their applicability is rather limited [7]. There is great interest in HAQN derivatives, because modest structural changes may induce drastic modifications in their absorption and fluorescence characteristics [8–10]. Among the HAQN family, carminic acid **2** (figure 1), extracted from cochineal insects (*Dactylopius coccus*) [11], and its derivative carmine **3** (figure 1) are frequently used as food biocolourants or for biological staining [12,13]. Since the end of the last century, great interest has been dedicated to carminic acid, due to its use as a natural food and cosmetic pigment. Various studies have investigated photodegradation [14], fluorescence properties in different solvents [15], reorientation dynamics [16], self-assembly [17] and acid–base behaviour [18]. However, the complete insolubility of carminic acid in apolar systems hampers its application, for example, in oil-based cosmetic and enamels formulations. The most common carminic acid derivatives are carmine **3** (figure 1) (resistant to light, heat and chemical oxidation, often it is more stable than other synthetic food colourants but unstable at low pH) [12] and some acid-stable derivatives such as 4-aminocarminic acid **4** (figure 1), still insoluble in organic solvents. Nowadays, the combination of dyes with polymers is a research field of great interest, and dyed polymers are becoming increasingly relevant as materials for several technical applications while also being a major part of everyday life. For instance, dye-containing polymers are widely applied in medicine, painting industries, analytics, and the search for non-toxic dyes may be of the utmost relevance in specific fields (i.e. children toys). A key issue in the production of coloured polymers is related to the dye's nature, in particular its affinity to the corresponding polymer [19]. In this paper, we propose the conversion of carminic acid into hydrophobic derivatives and their efficient incorporation in polyacrylate rubbers that can be used for producing, among other items, medical packaging, disposable hoses and squeeze toys.

## 2. Material and methods

### 2.1. Materials

Carminic acid was purchased from Sigma Aldrich and used without any further purification. *n*-Butyl acrylate (BA) from Sigma Aldrich was purified by freezing and thawing. 2,2′-Azobis-(isobutyronitrile) (AIBN, Sigma Aldrich) was recrystallized twice from methanol.

#### 2.1.1. General procedure for the esterification of carminic acid

Carminic acid (213** **mg, 0.433** **mmol) was dissolved in dry pyridine (4 ml) and acetic anhydride or pivaloyl chloride (1.25 equiv.) were added dropwise to the solution. The reaction was stirred at room temperature until there was complete consumption of the starting material. After completion, the reaction was diluted in dichloromethane and washed with 5% aqueous HCl. The organic layers were collected, dried over dry Na_2_SO_4_, filtered and evaporated to dryness.

Poly(butyl-acrylate) (PBA) specimens were obtained by bulk polymerization in a closed vial, at 80°C for 24 h, of a BA solution with 60** **ppm of an oil-soluble radical initiator, AIBN, and a dye concentration of 10**^−^**^3 ^M. Self-standing PBA specimens were obtained by adding a cross-linking agent, ethylene glycol dimethacrylate (EGDMA) (Sigma Aldrich), 4.5% in volume with respect to BA.

### 2.2. Computational details

All the investigated derivatives have been subjected to an initial systematic search within the torsion phase space of all rotable bonds by means of a classic molecular mechanics (MM)-based force field, i.e. MMFF94s [20]. The increment assigned to each torsion during the systematic search was 30°. The lowest lying energy isomeric structures resulting from the MM search have been subsequently optimized by DFT/B-P86/TZVP/COSMO protocol, that treats explicitly the whole electronic structure of the investigated species, and implicitly the solvent effects through the conductor-like screen model [21]. All calculations were performed using the turbomole [22] package for quantum chemistry. Excitation energies were computed in implicit dimethyl sulfoxide (DMSO) (*ε* = 46.826) at B3LYP [23,24] COSMO level in line with an exhaustive benchmark on HAQN [25]. Analytical gradients for the TDDFT excited state energy were computed using the EGRAD routine recently implemented [26] within the turbomole suite.

### 2.3. Characterization methods

^1^H-NMR and ^13^C-NMR spectra were obtained on a Bruker Avance 500 instrument operating at 500.13 and 125.76** **MHz for proton and carbon, respectively; NMR tubes were prepared using D_2_O as solvent for carminic acid and CDCl_3_ for the derivatives. Mass spectra were recorded on System Applied Biosystems MDS SCIEX instruments: Q TRAP, LC/MS/MS, turbo ion spray and Q STAR elite nanospray.

Differential scanning calorimetry (DSC) measurements were performed on a Mettler Toledo DSC1 STARe System at a constant nitrogen gas flow (50 cm^3^ min^−1^) from −130°C to 80°C.

Photoluminescence (PL) steady-state measurements were acquired by a 405 nm-pulsed diode laser (EPL-405 Edinburgh Instrument) and a polychromator equipped with a liquid nitrogen-cooled CCD detector. The same excitation with a polychromator equipped with a conventional time-correlated photon-counting board was used to acquire the time-resolved photoluminescence (TrPL) decays. A Varian Cary 50 spectrophotometer was employed for the absorption spectra. All of the measurements were carried out at room temperature.

## 3. Results and discussion

### 3.1. Carminic acid modification

**Figure 2.**
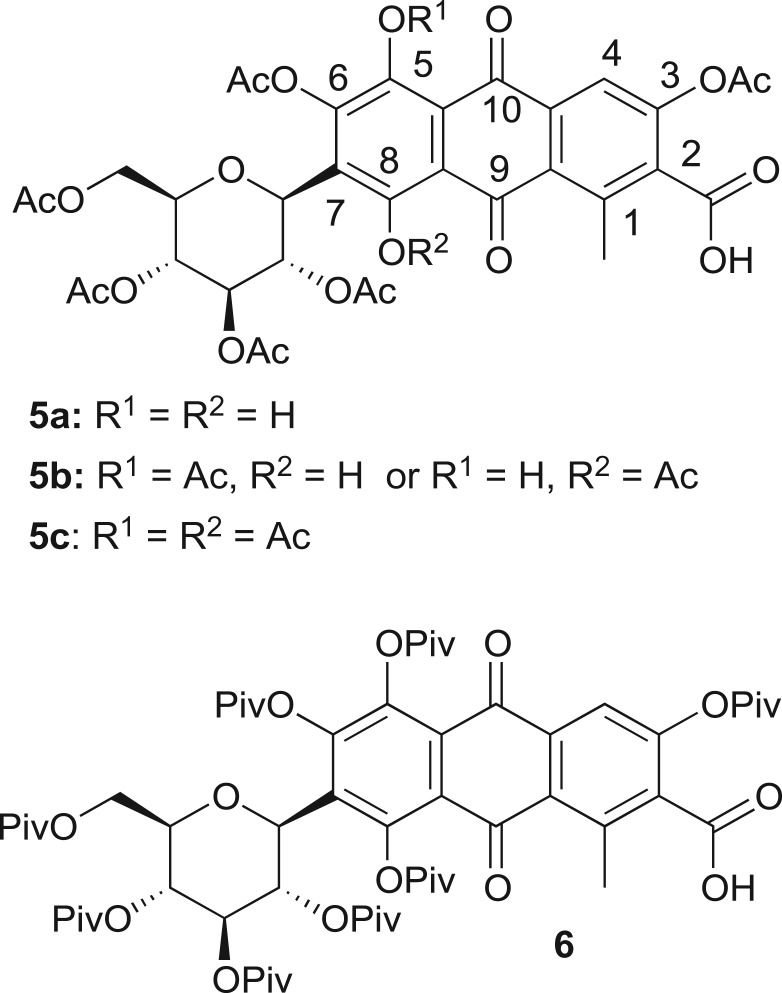
Products obtained from carminic acid upon esterification.

Esterification is a simple route for lowering hydrophilicity of polyhydroxylated compounds; thus, carminic acid was reacted with two common acylating agents, acetic anhydride [27] and pivaloyl chloride, affording compounds **5** and **6** (figure 2). The acetyl and pivaloyl groups were chosen taking into account the by-products, in case the ester derivatives of carminic acid undergo hydrolysis by enzymatic action upon ingestion. The reaction would result in the release of carminic acid together with acetic acid and pivalic acid, respectively. The acetic acid is a common product for several biological processes, while the pivalic acid has a very low toxicity (especially if considered in relation to the doses used for colouring) [28] and, for this reason, it is used by pharmaceutical companies for the synthesis of some pro-drugs that require a greater hydrophobicity (i.e. Dipivefrin™ for glaucoma treatment) [29].

After completion of the reaction, the organic extracts (dichloromethane) were collected and analysed by mass spectrometry, ^1^H and ^13^C-NMR (electronic supplementary material, figures S1–S5). Although the NMR spectra of the carminic acid completely agree with the ones reported by Schmitt *et al.* [30], those of the derivatives are not very revealing because they are dominated by the signals generated by the substituents [27]; furthermore, signals from the carminic core experience severe line-broadening making them barely detectable. The acetylation reaction afforded carminic acid as the main product acetylated at all positions but one (compound **5b**, figure 2), together with small amounts of peracetylated carminic acid (**5c**) and carminic acid lacking two acetyl groups (compound **5a**, figure 2). Unfortunately, these derivatives proved inseparable by chromatography; consequently, for the sake of simplicity, in the following this mix is named compound **5**. In anthraquinoid compounds, hydroxyl groups ***β*** to the carbonyl, that may occur at positions 1, 4, 5 and 8, are strongly involved in intramolecular hydrogen bonding; this feature strongly impacts on pKa values [18,29], chromogenic properties, and on the reactivity of the different phenolic hydroxyls [27,31,32]. In the case of carminic acid, the 5-OH and 8-OH groups are less reactive. On the contrary, the pivaloylation reaction afforded the fully esterified carminic derivative **6** (figure 2). The different reaction outcome can be ascribed to the bulky pivaloyl groups, that might disrupt planarity and weaken the hydrogen bonding responsible for structural rigidity and planarity [18]; as a result, hydroxyl groups increase their reactivity, allowing their exhaustive esterification.

**Figure 3.**
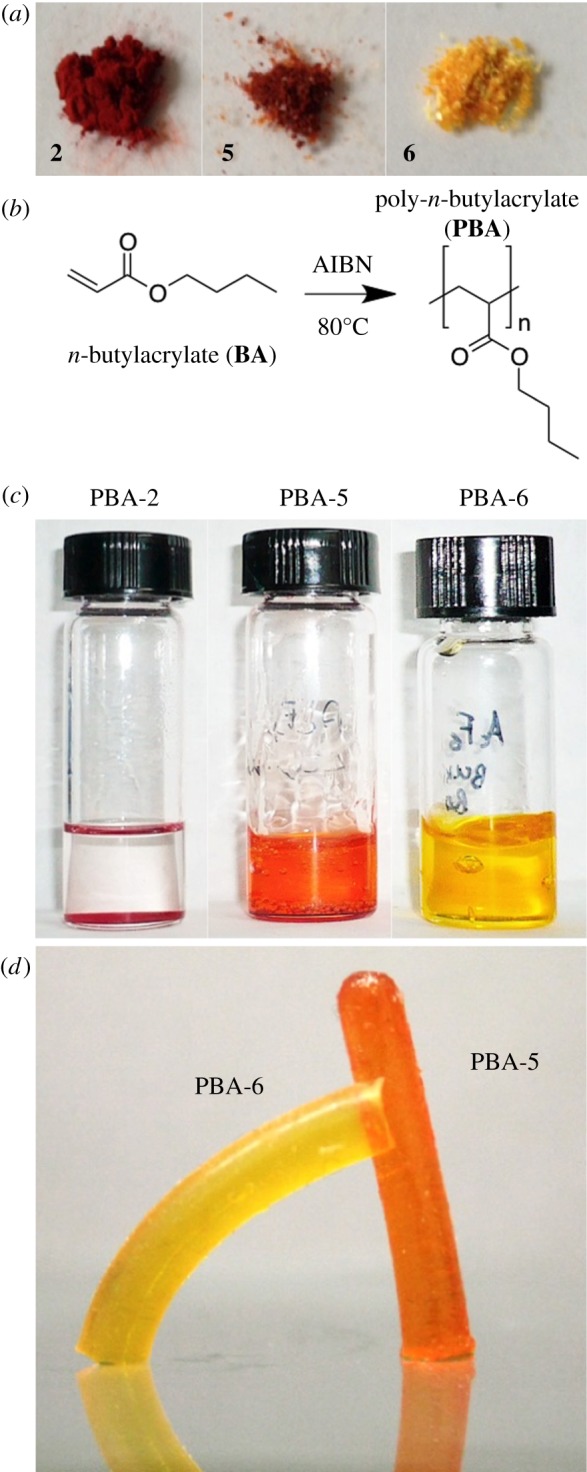
**(***a*) The colour of compounds **2**, **5** and **6** before polymer inclusion; (*b*) the polymerization reaction of BA to PBA; (*c*) the polymerization performed in the presence of compounds **2**, **5** and **6** (10–3 M in BA) affords PBA-2, PBA-5 and PBA-6, respectively; and (*d*) examples of objects obtained by polymerization of BA/EGDMA with derivatives **5** (right) and **6** (left).

DFT calculations confirm this hypothesis: the three-rings system experiences planarity disruption when switching from **2** to **6**: the torsion angle C2–C3–C6–C7, that allows fair evaluation of the possible deviation from planarity, goes from 0° in **2** to 5.9° in **6** (*vide infra* for more details). These observations are in agreement with previous reports [18,33]. The different chromogenic properties of compound **6** (figure 3*a*) further support this hypothesis: a shift from deep red for pure carminic acid (**2**) and acetyl derivative **5**, to orange–yellow of the pivaloyl derivative **6** is observed.

### 3.2. Carminic acid incorporation in organic polymers

Given the success in obtaining hydrophobic carminic acid derivatives, their incorporation into a polymer matrix was tested. Specifically, poly(*n*-butyl acrylate) has been selected as a reasonable representative of hydrophobic polymers. In fact, PBA has very interesting optical and mechanical properties, is highly transparent and colourless, and presents a low glass transition temperature (*T*_g_) (−50°C) [34,35]; furthermore, it can be synthesized by free radical bulk polymerization. Bulk-free radical polymerization is widely used for the production on an industrial scale of thick and large polyacrylate plates having excellent optical properties [36] without the need for organic solvents [37,38]. In order to efficiently include the dye in the polymer, the BA monomer must be able to dissolve it in a suitable concentration. The hydrophobic carminic acid derivatives were dissolved at a 10^–3^ M concentration in the monomer; the polymerization was initiated by an oil-soluble initiator (AIBN) through thermal activation. As a control, pristine carminic acid **2** was also used; however, being insoluble in organic systems, it also proved to be completely insoluble in the BA monomer. Polymerization of this heterogeneous system afforded a two-phase product (PBA-2, figure 3*c*); derivatives **5** and **6** are freely soluble in the organic monomer BA, and the polymerization proceeded efficiently affording a fully homogeneous dyed polymer.

As PBA has a very low *T*_g_, in order to fabricate elastomeric items with well-defined shapes a cross-linking agent is used, typically EGDMA. EDGMA presents two double bonds that can polymerize, thus after polymerization, an EGDMA moiety connects two polymer chains acting as a cross-link. By varying the amount of cross-linking agent, it is possible to tune the final mechanical properties, such as impact strength and abrasion resistance.

As depicted in figure 3*d*, massive PBA objects were fabricated by polymerization of BA in the presence of 4.5% EGDMA (v/v) and carminic acid derivatives **5** and **6**.

### 3.3. Physico-chemical characterizations

The polymers PBA-5 and PBA-6 and copolymers (PBA/EGDMA-5 and PBA/EGDMA-6) were characterized for their bulk *T*_g_ by DSC, and for their spectroscopic properties, and compared with neat derivatives **5** and **6**.

**Figure 4.**
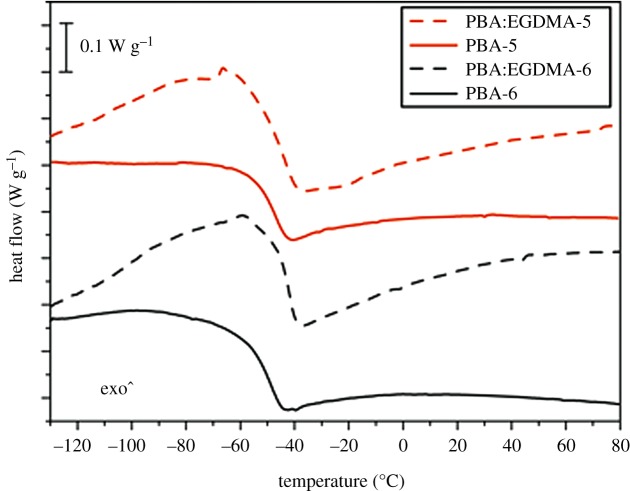
DSC analysis for polymers in the presence (PBA–EGDMA) and in the absence of EGDMA (PBA), including carminic acid esters **5** and **6**.

All the PBA samples, cross-linked or not, present in the DSC traces a *T*_g_ around −50°C, which is the usual *T*_g_ value for high molecular weight PBA (figure 4). Thus, the inclusion of carminic acid derivatives does not significantly alter polymerization kinetics and polymer thermal properties. Also, the amount of EGDMA used for cross-linking does not affect the local dynamics of the polymer chains.

Absorption, steady-state PL and TrPL investigation were also performed in order to deepen the physics and spectroscopic properties of carminic acid and their derivatives in pure forms, and were included in the PBA matrix. Since, as clearly visible in figure 3, the presence of EGDMA does not alter the colour of PBA samples, only samples without cross-linker are analysed.

**Figure 5.**
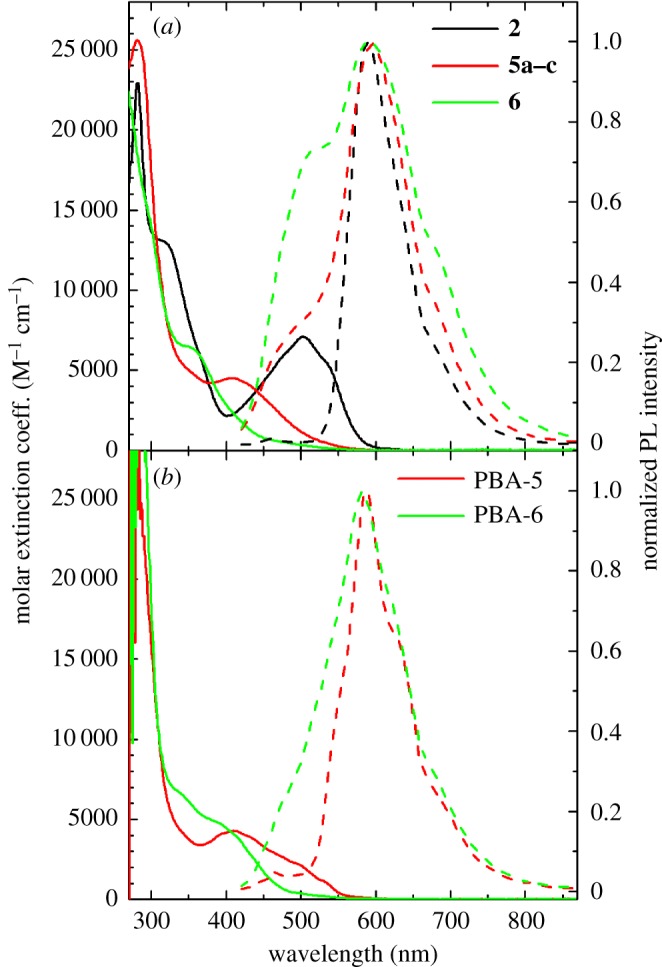
(*a*) Molar extinction coefficient (solid lines) and PL (dashed lines) spectra acquired for compounds **2** (in water), **5** and **6** (in DMSO). (*b*) Molar extinction coefficient (solid lines) and PL (dashed lines) spectra acquired for PBA-5 and PBA-6.

The molar extinction coefficient estimated for compounds **2**, **5** and **6** in solution (**2** in water and **5** and **6** in DMSO solution) are shown in figure 5*a* (solid line). Carminic acid **2** absorption spectra, in accordance with the literature data, shows a band peak at about 500** **nm, which confers the characteristic red colour, and a more intense band at about 290** **nm [39,40]. On the other hand, derivatives **5** and **6**, characterized by a similar band at 300** **nm when compared with carminic acid **2**, show a blue-shifted band (hypsochromic shift) in the visible range. Indeed, molecule 5 has a band peak at about 410** **nm, whereas only a shoulder at 360** **nm can be detected for compound **6**. These observations are in perfect agreement with the absorption behaviour of anthraquinone dyes: an hypsochromic effect is usually observed when derivatizations at position 5 and 8, with substituents that are not able to remove hydrogen bonding with 9 and 10 carbonyls, cause a reduction in conjugation of the chromophore, resulting in absorption at shorter wavelengths [41].

The steady-state PL spectra of the compounds in solution are also reported in figure 5*a* (dashed lines). In agreement with previous works [31,32], the PL spectrum of carminic acid **2** peaks at 610** **nm with a good mirror symmetry compared with the absorption band. On the other hand, the PL spectra of the derivatives are dominated by the same emission at 610** **nm, but with the superimposition of another band peaking at about 500** **nm. We interpreted this latter band as the emission directly related to the absorption bands at 410 and 360** **nm of derivatives **5** and **6**. The emission at 610** **nm observed in both compounds **5** and **6** can instead be ascribed to the contribution of some residual carminic acid. Indeed, because the emission of derivatives **5** and **6** is perfectly resonant with the first absorption band of the carminic acid, the energy transfer between the newly synthesized compounds to this residual moiety is expected to be especially efficient even when the concentration of the impurity is very low. It is worth noting that in absorption spectra of compounds **5** and **6**, shown in figure 5*a*, the contribution of carminic acid is not detectable, and therefore it does not affect the colour of compounds **5** and **6**.

To investigate the effect of incorporation in organic polymers, we report in figure 5*b* the absorption and steady-state spectra for PBA-5 and PBA-6. Both derivatives exhibit almost the same absorption spectra, in terms of shape and molar extinction coefficient, in respect of the corresponding compounds in solution, suggesting that PBA matrices do not affect the absorption properties. Different results were obtained for the corresponding PL spectra. Indeed, as shown in figure 5*b*, the PL spectra of PBA-5 and PBA-6 are dominated by the same emission band peak at 610** **nm when compared with the PL spectra of **5** and **6** in DMSO solution, but the emission band at 500** **nm is almost absent. This emission, well-rendered in samples in **5** and **6** in solution, disappears when **5** is included in PBA and just barely noticeable in the tail emission of PBA-6. This finding is not due to the incorporation into polymer, but can be ascribed to the higher concentration of compounds **5** and **6** in polymer than in solution. Indeed, a dye concentration of 10**^−^**^4^ M was considered for compounds in DMSO solution instead of 10**^−^**^3^ M in the polymer objects.

Finally, the TrPL investigation reported in the electronic supplementary material, figures S6 and S7, reveals that carminic acid and its hydrophobic derivatives (in DMSO solution and in PBA polymer) are characterized by lifetime decays over the range of a few nanoseconds. These findings suggest that the emission occurs from a singlet–singlet allowed transition, in agreement with the computational analysis discussed in the next paragraph.

In order to determine the commercial colour name of the obtained hydrophobic compounds in PBA, for possible applications in cosmetics and toys production, we carried out colorimeters measures. We found a ‘bright red orange’ and ‘zinc yellow’ colour according to the classic RAL system for PBA-5 and PBA-6, respectively. In particular, we estimated, in CIE-L*a*b* colour space: L* = 53.14, a* = 55.27, b* = 61.25 for PBA-5, and L* = 87.97, a* = −3.30, b* = 84.49 for PBA-6.

### 3.4. Computational studies

In order to better characterize the structural and spectroscopic properties of hydrophobic carminic acid derivatives, MM, DFT and TDDFT calculations were performed. QM calculations have proved strongly supportive of the disruption of three-rings system planarity in pivaloyl carminic derivative (see earlier).

**Figure 6.**
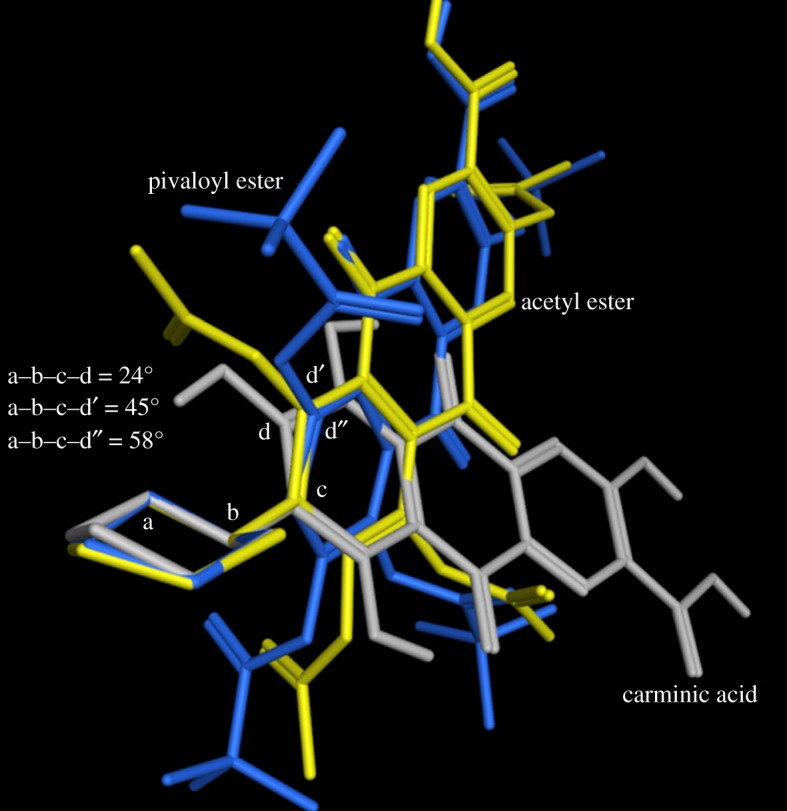
Overlap of ground-state structures of **2**, **5c** and **6**. Displayed values illustrate quantitatively the extent of intramolecular rearrangement occurring upon esterification of carminic acid and the *R* size increasing as well.

Moreover, from ground-state investigation, a clear trend has emerged regarding the structural intramolecular rearrangement brought about by the esterification process with increasing the *R* size. Indeed, the mutual orientation between the poly-aromatic core and the sugar skeleton varies significantly and steadily upon going from carminic to pivaloyl derivative passing through the acetylated derivative. A torsion angle representing the reciprocal spatial disposition of the two groups (defined by the letters a–b–c–d in figure 6) has been measured on the ground-state structures of **2** (carminic), **5c** (peracetyl derivative) and **6** (perpivaloylated derivative), and results clearly show an approximate increment of approximately 20° on switching from **2** to **5c**. The target torsion angles increase by 13° when the *R* size gets larger (i.e. from **5c** to **6**). In the case of **5b** (*R*^2^ = H), the equivalent dihedral is 46.7°.

**Figure 7.**
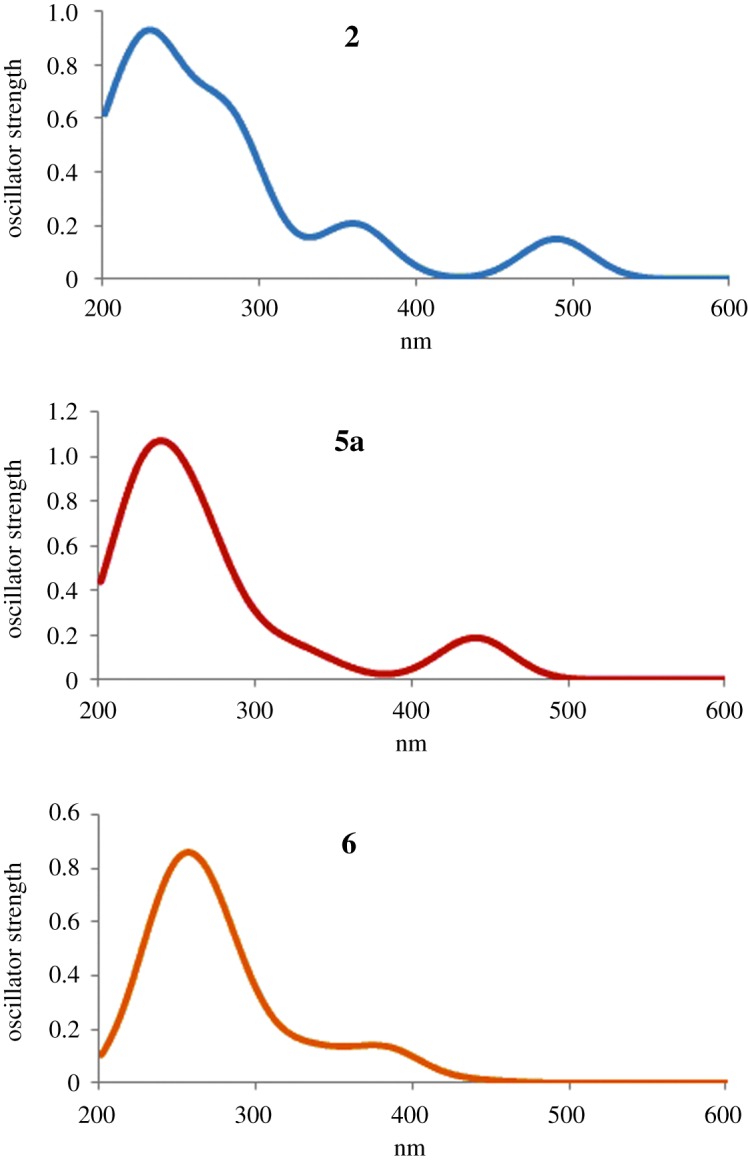
TDDFT absorption spectra for **2**, **5a** and **6**. Excitation energies in nanometres.

The **2**, **5b** (*R*^2^ = H) and **6** optimized structures have been used to evaluate the electronic spectra at TDDFT level, adopting B3-LYP functional methods to better calculate excitation energies (figure 7). Using the same computational setting, the electronic spectra of the HAQN obtained removing the glucosidal part have been computed. In the electronic supplementary material, table S1 reports the most relevant results of this investigation. The level of theory adopted reproduces the experimental excitation energies for the first most intense absorption with an average uncertainty of 30** **nm.

**Figure 8.**
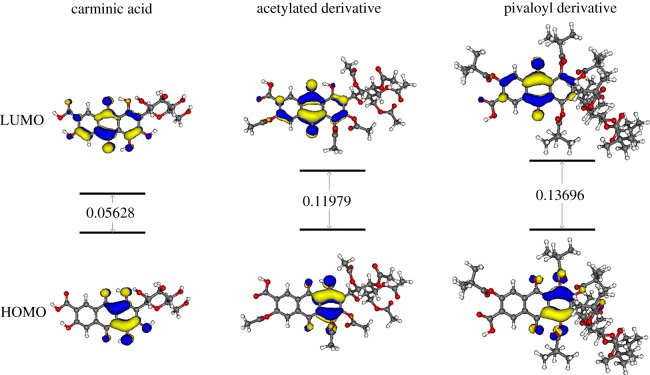
Isodensity plots of HOMO and LUMO of **2**, **5b** and **6** (isosurface at 0.04 atomic units) involved in the most intense low-energy single excitations (S1 for **2** and **5b**, S3 for **6**). The HOMO/LUMO gap is reported in hartree.

Going from **2** to **6**, the trend of the blue shift for the first most intense absorption reproduces the experimental spectra well. The chemical nature of the *R* groups on the HAQN modulates the excitation energies of the system considered. The main mono-electron transition of these absorptions is the HOMO → LUMO that corresponds to the π → π* transition of the HAQN portion (figure 8).

The aforementioned observation is also compatible with the nearly identical excitation energy values associated with the first most intense transition in both the HAQN portions and in the complete systems. Going from **2** to **6**, the HOMO, that is mainly localized on the ring adjacent to the sugar group, results in being stabilized. As a consequence, the HOMO–LUMO gap gets wider, which is reflected in the blue shift of the absorption bands. By TDDFT geometry optimization of the S1 state, we predict emission energies at 613** **nm for **2**, 598** **nm for **5b**, and 569** **nm for **6**.

## 4. Conclusion

New hydrophobic derivatives of carminic acid, a widely used food dye, have been efficiently synthesized for the first time and included in polymeric hydrophobic matrices; moreover, the computational investigations allowed rationalizing of experimental data, elucidating some stereoelectronic factors underlying the spectral features of synthesized carminic acid derivatives. The proposed work suggests a new and more bio-friendly approach in dyeing rubbers, replacing the use of azo dyes. Azo dyes are widely used for colouring a plethora of consumer goods, from leather to food, and from cosmetics to toys [42,43]. Especially in the case of toddlers, dolls, small animals and objects for playing with are very soft and thus made of rubber, and many times they are not only handled but are put in the mouth, with the risk that children ingest the colourants. Although azo dyes offer a wide spectrum of colours and good resistance to degradation under aerobic conditions, they can be reduced to aromatic amines under anaerobic conditions [44,45]. The carcinogenic effect of such aromatic amines on humans [46–49] may limit their usage. As the main routes of exposure of consumers to azo dyes are dermal absorption and oral ingestion, replacing azo-based dyes with the hydrophobic derivatives of carminic acid might lead to a new class of dyes for the polymer industry, particularly for dermocosmesis and child-oriented goods.

## Supplementary Material

Figures S1 - S4 and Table S1

## Data accessibility

The datasets supporting this article have been uploaded as part of the electronic supplementary material: https://figshare.com/s/063c5c84c2547155963c.
