# Supplementary material for: Towards hydrophobic carminic acid derivatives and their incorporation in polyacrylates
Source: R Soc Open Sci. 2018 Jul 4;5(7):172399. doi: 10.1098/rsos.172399 (PMC6083691; doi:10.1098/rsos.172399)
Supplement: Figures S1 - S4 and Table S1 [file rsos172399supp1.docx]

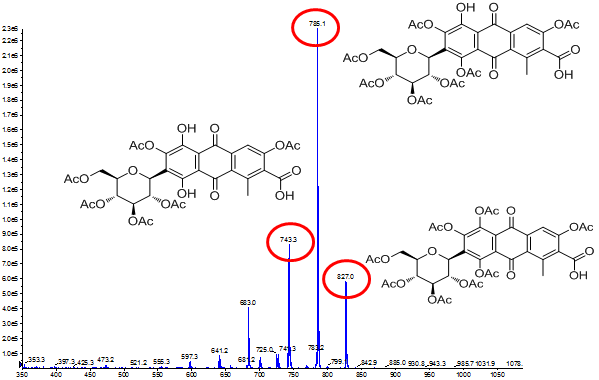


**Figure1_ESM.** Mass spectrum of the acetylated products **5a-c**.


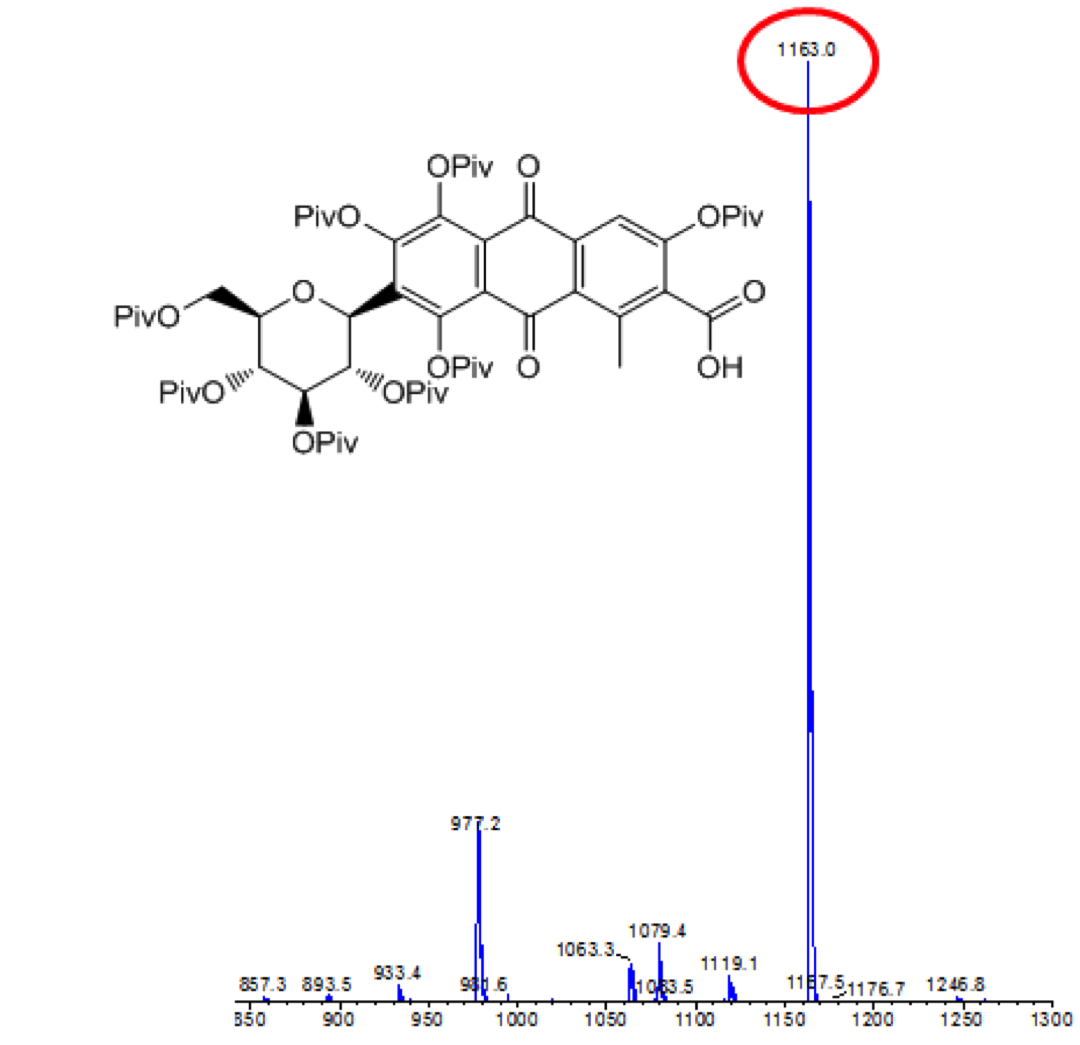


**Figure 2_ESM.** Mass pectrum of the pivaloylated product **6**.

**
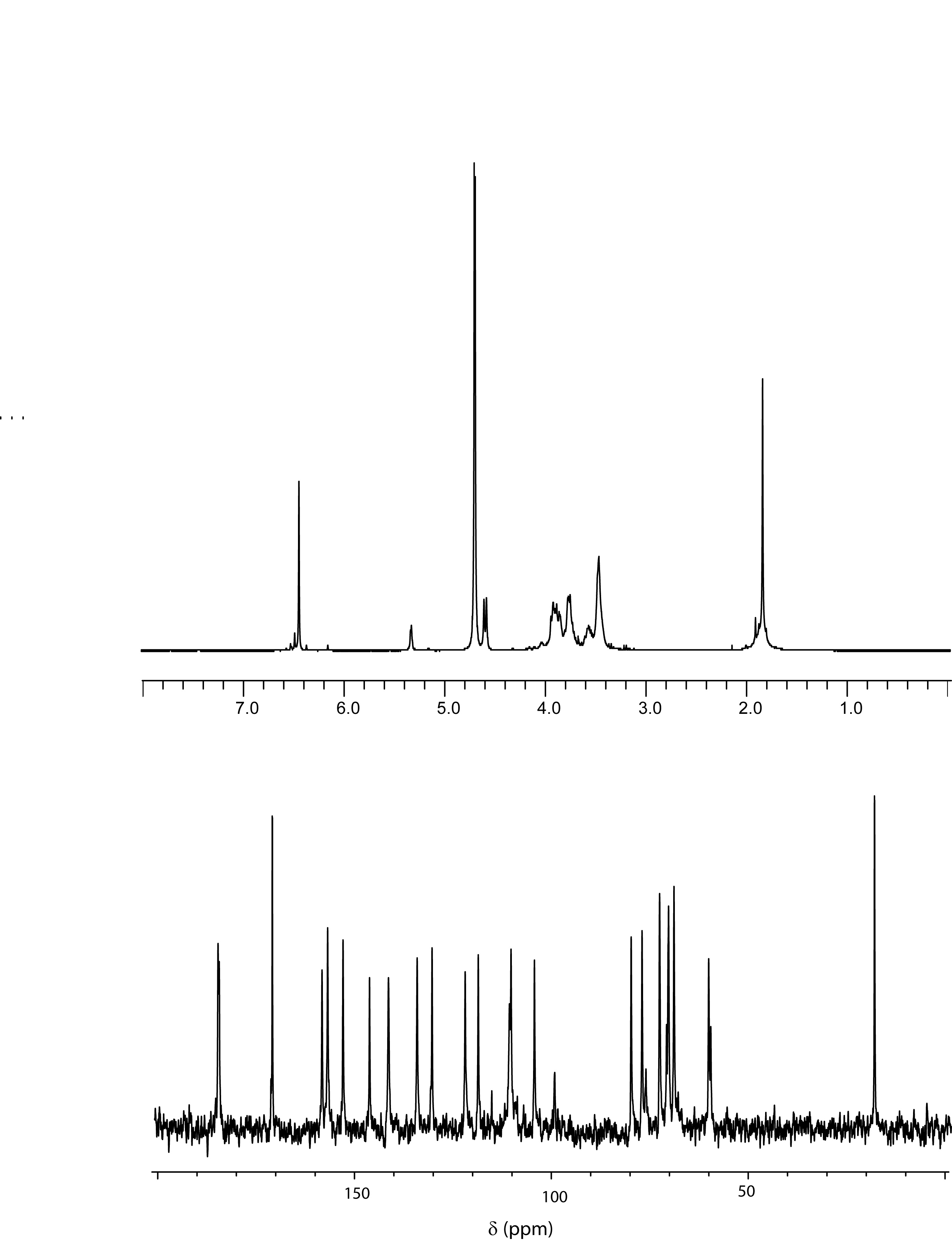
**

**Figure3_ESM.** ^1^H NMR (top) and ^13^C NMR bottom spectra of **3.**


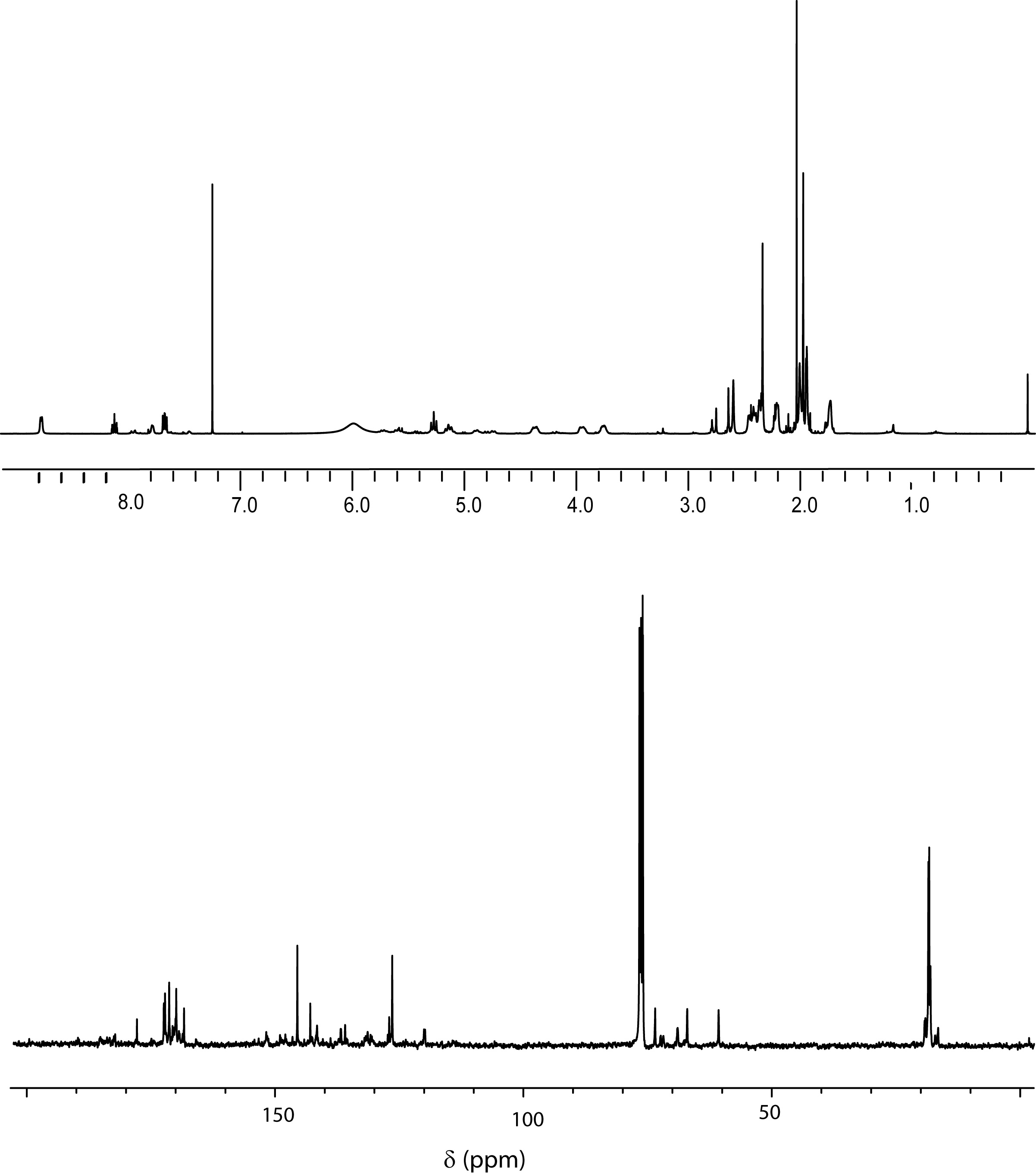


**Figure4_ESM.** ^1^H NMR (top) and ^13^C NMR bottom spectra of **5a-c.**


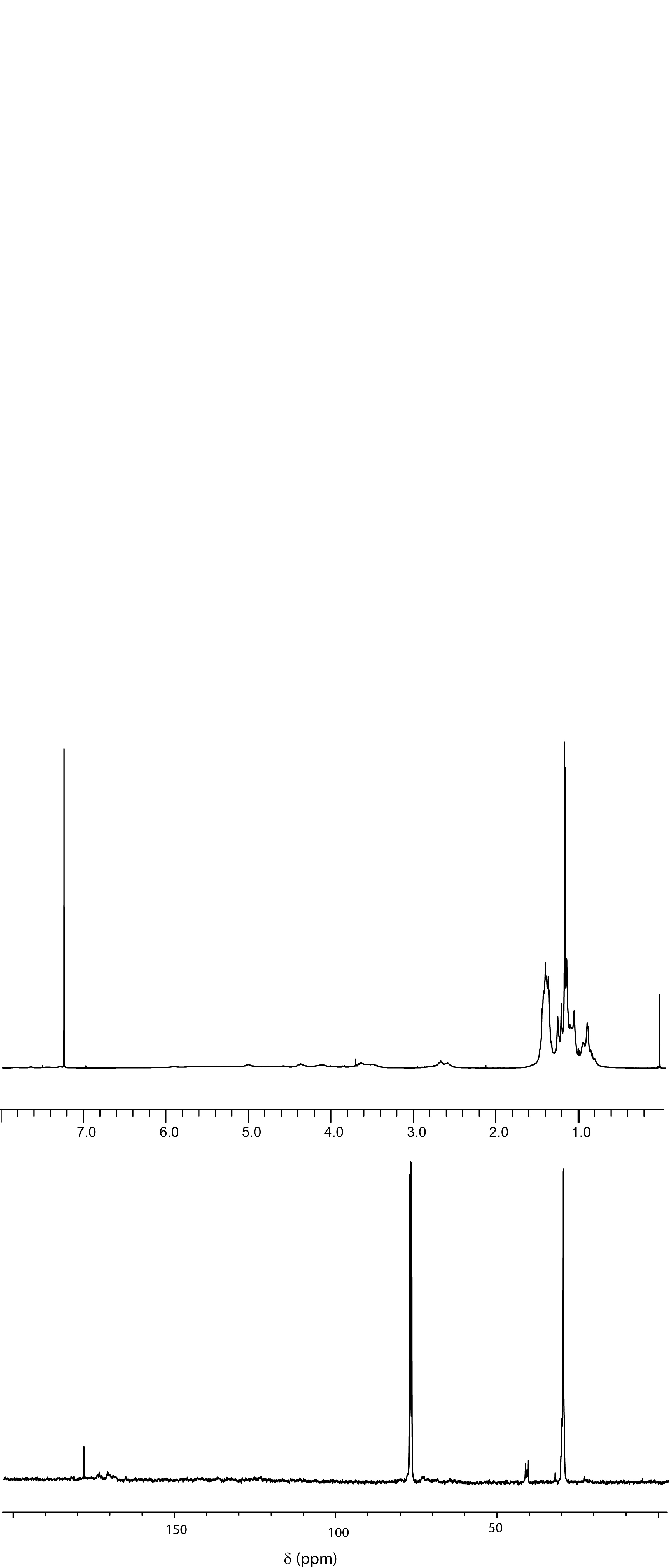


**Figure5_ESM.** ^1^H NMR (top) and ^13^C NMR bottom spectra of **6.**

TrPL decays at 600nm emission acquired for carminic acid **2** in water, acetylated derivatives **5a-c** and pivaloyl derivative **6** in DMSO solution are reported in Fig. S1. The corresponding TrPL decays acquired for **PBA-5a-c** and **PBA-6 in PBS** polymer are shown in Fig. S2. All the decay curves can manly described as a single exponential function, except for some sample **5a-c** (Fig. S1b) and **PBA-6** (Fig. S2b), in which a little deviation was observed. The lifetimes (τ) estimated from data of Fig. S1 and Fig. S2 are reported in Table S1. The values fall in the range of ns (from 1.5 to 8.3 ns), suggesting singlet-singlet transitions.


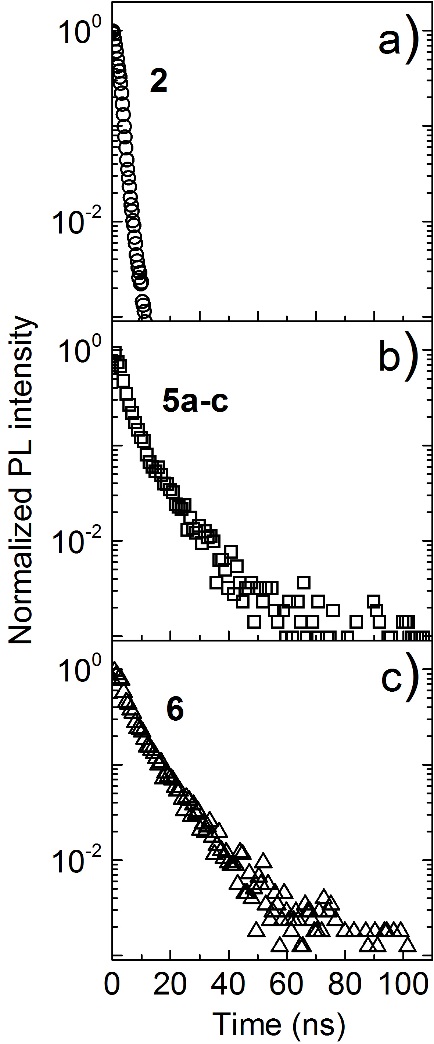


**Figure6_ESM** TrPL decays at 600 nm emissions acquired for: a) **2**, b) **5a-c** and c) **6**.


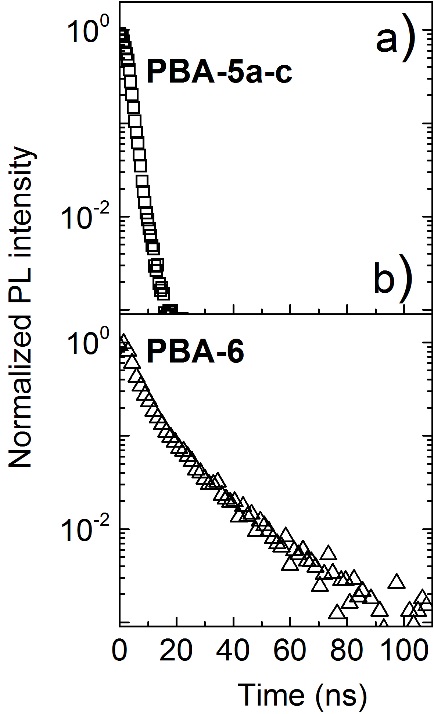


**Figure7_ESM**) TrPL decays at 600 nm emissions acquired for: a) **PBA-5a-c** and b) **PBA-6**.

**Table 1_ESM**

**2**, **5b** and **6** computed electronic spectra. For each system are reported the excitation energies (nm) in nm of the most intense transitions, the corresponding oscillator strength (f) and the main 1-electron transitions (1e). The lowest most intense experimental transition (exp) is also reported for the sake of comparison. nm_HAQN_ ,f_HAQN_ and 1e_HAQN_ are the excitation energies, oscillator strength and 1-electron transition for the sole HAQN portion.

|  |  | **nm** | **f** | **1e** | **exp** | **nm_HAQN_** | **f_HAQN_** | **1e_HAQN_** | |
| --- | --- | --- | --- | --- | --- | --- | --- | --- | --- |
| **2** | S_1_ | 492 | 0.19 | HOMO→LUMO | 503 | 491 | 0.13 | HOMO→LUMO | |
|  | S_4_ | 366 | 0.16 | HOMO-2→LUMO+1 (87%) |  |  |  |  | |
|  | S_27_ | 230 | 0.36 | HOMO→LUMO+3 (45%)  HOMO-7→LUMO+1 (35%) |  |  |  |  | |
|  | S_48_ | 196 | 0.17 | HOMO-2→LUMO+3 (47%)  HOMO-21→LUMO (13%) |  |  |  |  | |
| **5b** | S_1_ | 444 | 0.15 | HOMO→LUMO (79%)  HOMO-3→LUMO (16%) | 408 | 441 | 0.11 | HOMO→LUMO (80%)  HOMO-3→LUMO (12%) | |
|  | S_20_ | 269 | 0.19 | HOMO-2→LUMO (60%)  HOMO-1→LUMO+1 (11%) |  |  |  |  | |
|  | S_43_ | 228 | 0.20 | HOMO-2→LUMO+2 (34%) |  |  |  |  | |
|  | S_50_ | 220 | 0.20 | HOMO-12→LUMO (20%) |  |  |  |  | |
| **6** | S_1_ | 436 | 0.01 | HOMO-5→LUMO (55%)  HOMO→LUMO (34%) |  |  |  |  | |
|  | S_3_ | 389 | 0.11 | HOMO→LUMO (49%)  HOMO-5→LUMO (27%) | 342 | 376 | 0.11 | HOMO→LUMO (60%)  HOMO-1→LUMO (32%) | |
|  | S_21_ | 270 | 0.13 | HOMO-22→LUMO (48%)  HOMO-3→LUMO+1 (33%) |  |  |  |  | |
|  | S_31_ | 254 | 0.14 | HOMO-11→LUMO+1 (37%)  HOMO-7→LUMO+1 (10%) |  |  |  |  | |
|  | S_47_ | 238 | 0.23 | HOMO-12→LUMO+1 (49%) |  |  |  |  |  |

**Table 2_ESM**

**2**, **5b** and **6** computed electronic spectra. For each system are reported the excitation energies (nm) in nm of the most intense transitions, the corresponding oscillator strength (f) and the main 1-electron transitions (1e). The lowest most intense experimental transition (exp) is also reported for the sake of comparison. nm_HAQN_ ,f_HAQN_ and 1e_HAQN_ are the excitation energies, oscillator strength and 1-electron transition for the sole HAQN portion.

|  |  | **nm** | **f** | **1e** | **exp** | **nm_HAQN_** | **f_HAQN_** | **1e_HAQN_** | |
| --- | --- | --- | --- | --- | --- | --- | --- | --- | --- |
| **2** | S_1_ | 492 | 0.19 | HOMO→LUMO | 503 | 491 | 0.13 | HOMO→LUMO | |
|  | S_4_ | 366 | 0.16 | HOMO-2→LUMO+1 (87%) |  |  |  |  | |
|  | S_27_ | 230 | 0.36 | HOMO→LUMO+3 (45%)  HOMO-7→LUMO+1 (35%) |  |  |  |  | |
|  | S_48_ | 196 | 0.17 | HOMO-2→LUMO+3 (47%)  HOMO-21→LUMO (13%) |  |  |  |  | |
| **5b** | S_1_ | 444 | 0.15 | HOMO→LUMO (79%)  HOMO-3→LUMO (16%) | 408 | 441 | 0.11 | HOMO→LUMO (80%)  HOMO-3→LUMO (12%) | |
|  | S_20_ | 269 | 0.19 | HOMO-2→LUMO (60%)  HOMO-1→LUMO+1 (11%) |  |  |  |  | |
|  | S_43_ | 228 | 0.20 | HOMO-2→LUMO+2 (34%) |  |  |  |  | |
|  | S_50_ | 220 | 0.20 | HOMO-12→LUMO (20%) |  |  |  |  | |
| **6** | S_1_ | 436 | 0.01 | HOMO-5→LUMO (55%)  HOMO→LUMO (34%) |  |  |  |  | |
|  | S_3_ | 389 | 0.11 | HOMO→LUMO (49%)  HOMO-5→LUMO (27%) | 342 | 376 | 0.11 | HOMO→LUMO (60%)  HOMO-1→LUMO (32%) | |
|  | S_21_ | 270 | 0.13 | HOMO-22→LUMO (48%)  HOMO-3→LUMO+1 (33%) |  |  |  |  | |
|  | S_31_ | 254 | 0.14 | HOMO-11→LUMO+1 (37%)  HOMO-7→LUMO+1 (10%) |  |  |  |  | |
|  | S_47_ | 238 | 0.23 | HOMO-12→LUMO+1 (49%) |  |  |  |  |  |
